# Environmental Degradation of Footbed Materials Under Different Conditions

**DOI:** 10.3390/polym17233134

**Published:** 2025-11-25

**Authors:** Asis Patnaik, Sudhakar Muniyasamy, Ashvani Goyal

**Affiliations:** 1 Department of Clothing and Textile Technology, Faculty of Engineering and the Built Environment, Cape Peninsula University of Technology, Cape Town 7535, South Africa; 2 CSIR Chemicals, Advanced Polymer Composites, Pretoria 0001, South Africa; 3 Department of Chemistry, Nelson Mandela University, Gqeberha 6031, South Africa; 4 Department of Textile Technology, Technological Institute of Textile & Sciences, Bhiwani 127021, India; ashvanigoyal@titsbhiwani.ac.in

**Keywords:** artificial weathering, biodegradation, environmental degradation, footbed materials fragmentation

## Abstract

Different types of polymeric materials are used as footbeds in shoes. Environmental degradation behavior of polymeric footbed materials is an important parameter for understanding materials’ environmental footprint. Most of the previous studies focus on geotextiles, polymeric insulation materials, and exposure behaviors that are not the same due to the nature of applications of geotextiles and insulations being completely different from the footbeds. There is a lack of studies to understand artificial weathering, the influence of physical–chemical factors, and the subsequent behavior of different types of footbeds. In this paper, we have selected three needle-punched nonwoven footbed materials and studied their environmental degradation behavior by subjecting them to artificial weathering using different exposure durations, viz. 120 h, 240 h, and 360 h. The physical–chemical properties of polymeric footbed materials were characterized by Fourier-Transform Infrared Spectroscopy (FTIR), Differential Scanning Calorimetry (DSC), and thermogravimetric analysis (TGA). The selected polymeric footbed materials were made from recycled polyester (RPET), hemp, and shoddy fibers. Furthermore, the RPET footbed was tested for biodegradation in soil and compost conditions for 120 days. The footbed materials were also tested for physical and performance (tensile and abrasion resistance) properties. Hemp footbed materials undergo abiotic degradation after 120 h, but in the case of RPET, it undergoes abiotic degradation after 360 h, resulting in a fragmentation process due to synergistic effects of chemical and hydrolytic degradations. From the DSC results, RPET undergoes a slight thermal transition under abiotic degradation after 360 h, indicating that environmental abiotic factors influence degradation behavior. The tensile and abrasion resistance properties of RPET were the highest, followed by hemp and shoddy materials. The tensile strength range of the materials was between 50.74 and 851.44 N. The weight loss range after abrasion resistance was 0.016–0.014%. From the RPET biodegradation test in soil and compost conditions, the evolved CO_2_ was 20% and 59%, respectively, after 110 days. The DSC and TGA results indicate that the hemp footbed materials have a higher rate of abiotic degradation as compared to the RPET and shoddy footbed materials. From the RPET biodegradation test in soil and compost conditions, the CO_2_ degradation values were 20% and 59%, respectively. The obtained degradation results indicate that the synergistic effect of abiotic and biotic conditions greatly influences footbed materials’ biodegradation under natural environmental conditions.

## 1. Introduction

With the increasing focus on the use of sustainable nonwoven materials for various applications, including footwear, many alternative materials are used in footwear, particularly covering the footbed part (or insole), which is the nonwoven component. The footbed is one of the main parts of the footwear where the feet rest. These footbeds are discarded after use, or they are completely dumped off along with the shoe if the shoe is worn out or out of shape. The discarded materials are exposed to natural weather conditions, which take into consideration ultraviolet light (UV) from the sun, temperature, humidity, dew, and rain.

One of the ways to simulate material degradation behavior is by artificial weathering, which considers all environmental abiotic factors like UV, temperature, humidity, dew, and rain. The results from such studies provide important information about material degradation behavior, which is mostly overlooked in the material selection and design phases of product manufacturing. In a paper, the authors studied the durability and sustainability of shoe insoles made from needle-punched nonwovens by using recycled polyester (RPET) fibers [1]. The authors reported that the insole materials were durable and sustainable, but no data was reported covering the sustainable part and end-of-life impact of the materials [1].

The biodegradability and composting properties of footwear materials were studied by aerobic biodegradability tests in soil, and the degradation behavior was reported [2]. The authors tested various properties, including morphological, abrasion resistance, and thermal properties, before and after exposing them to the simulated soil environment. The authors suggested the importance of using sustainable and biodegradable materials in the design and development of footwear. Fakayode et al. 2025 studied various shoe brands and their insoles [3]. The insoles were tested for the thermal stability of the constituent polymers. They reported that insole thermal degradation varies and generally degrades around 600 °C [3].

Rahmouni et al. 2025 reported a nonwoven polymeric material developed from polyvinyl chloride (PVC) waste and PET for artificial leather applications with the aim of addressing environmental footprint and sustainability challenges [4]. The authors evaluated various physico-mechanical properties, such as thickness, weight/unit area, tensile strength, elongation, tear strength, and abrasion resistance. With the increasing PVC powder proportion, the physico-mechanical properties of the recycled nonwoven fabrics were significantly increased. Furthermore, a higher PET fiber percentage (72%) significantly improved the abrasion resistance related to weight loss and visual appearance of the fabrics.

Messaoud et al. 2013 discussed a method of producing an RPET insole laminated with different textile polymeric materials [5]. The authors further characterized the physical and compression properties of the insole structure. It was reported that insoles laminated with hemp woven fabric have the highest water absorbency, thermal conductivity, and the coolest touch effect compared to nonwoven made of a blend of polymeric polyester and viscose fibers.

The performance of different types of airlaid nonwovens made from short cellulose fibrils and superabsorbent (SAP) polymers was tested in terms of moisture absorption property from air with high relative humidity in a climatic chamber [6]. The test simulated the temperature and humidity conditions that occur inside rubber protective footwear. These nonwovens were manufactured for footwear insole applications, focusing on improving comfort properties. The authors reported that insoles with SAP dehumidified the air more effectively than non-SAP insoles. These results showed SAP can be used in footwear insoles in combination with nonwovens to improve wearer comfort.

In a study, the authors report on the development of a composite resin consisting of thermoregulatory phase-change material (PCM) [7]. The objective of this development was to alter the temperature of orthopedic insoles to improve wearer thermal comfort. The authors found that the thermal comfort of the insole depended upon the quantity of PCM, but at the same time, it resulted in poor mechanical properties.

Gottfridsson and Zhang (2015) studied shoe consumption in Sweden and its environmental impacts between 2000 and 2010 with a model considering life cycle assessment and product and material flow analyses [8]. The shoes contributing to the environmental impact in 2010 were leather shoes, up to 50%; rubber and plastic shoes, up to 26%; and textile shoes, up to 17%. The authors reported that material production corresponds to the highest impact, with 80% of the total life cycle, and natural textiles and wood materials are preferable compared to leather, rubber, and synthetic fiber-based polymeric materials regarding environmental impact. In another study, Zottin (2019) also agreed with similar findings, i.e., conventional footwear using synthetic polymeric materials, requiring large water and energy usage, contributing to greenhouse gas (GHG) emissions and environmental pollution [9].

In another study, the authors reported the performance and soil degradation of needle-punched nonwoven mulches made from natural fibers and poly lactic acid (PLA) for agricultural applications [10]. The nonwovens were made from jute, hemp, viscose, and PLA with varying area weights. There were no significant changes in mass per unit area, thickness, air permeability, and tensile properties of nonwovens, and they exhibited no degradation during the 300 days of exposure to environmental conditions.

Aparicio-Ardila et al. 2021 studied the degradation behavior of polypropylene geotextiles exposed to natural weathering between 1 and 3 years [11]. The results showed a decrease in the physical and mechanical properties of the geotextiles linked to the exposure duration of three years, showing a lesser value compared to one-year values. Similar results were reported by other researchers [12].

Material biodegradation in different environmental conditions also plays a significant role in understanding the environmental footprint of the materials. The biodegradation behavior of waste wool and RPET-based nonwoven was studied in aqueous and soil conditions [13]. For waste wool fibers, the authors reported 90% and 60% biodegradation in soil and aqueous media conditions for a 100-day test. The biodegradation results were reported in terms of carbon dioxide (CO_2_) emission. While maintaining the same conditions for REPT, the authors reported 20% and 10% biodegradation in soil and aqueous media conditions. In another study, the authors reported the biodegradation behavior of waste wool and RPET for building industry applications. They achieved 65–70% biodegradation for waste wool/RPET mats tested for 50 days in a compost [14].

From the above literature scan, there are three major research gaps on the environmental degradation of footbed materials under different conditions. *The first gap* is the changes in the physical–chemical parameters that describe changes in the material’s structure and surface properties during the degradation phases, which play an important role in environmental analysis. How do physical–chemical parameters change after artificial weathering? *The second gap* is the types of polymeric footbed materials used for degradation studies. It can be seen from the above literature scan that only a few studies were published covering the environmental degradation behavior of nonwoven footbeds. Most of the previous studies focus on geotextiles, insulation materials, and exposure behaviors that are not the same due to the nature of applications of geotextiles and insulations being completely different from the footbeds. *The third gap* is the duration of the degradation test, influence of humidity/dew/rainwater simulating a real environment, and types of degradation testing (artificial weathering, in soil, natural sunlight, and laboratory conditions). We have considered three different durations, 120 h, 240 h, and 360 h, for the abiotic degradation test. The 120 h accelerated test is approximately equivalent to 55–60 days of real conditions in South Africa. We have tested footbed materials from 55 days (120 h)–180 days (360 h) in the accelerated weathering chambers in the presence of humidity/dew/rainwater, simulating actual weathering conditions. Furthermore, with growing concern regarding the use of different polymeric materials, sustainable issues, and their environmental footprint, we have tested one of the most widely used polymeric materials, RPET, for biodegradation tests in soil and compost conditions for 120 days.

The properties of the materials were characterized by Fourier-Transform Infrared Spectroscopy (FTIR), Differential Scanning Calorimetry (DSC), and thermogravimetric analysis (TGA). One of the footbed materials, RPET, was tested for the biodegradation test in soil and compost conditions for 120 days. These materials were also tested for physical (thickness and area weight) and performance (tensile and abrasion resistance) properties.

## 2. Materials and Methods

### 2.1. Materials

**Figure 1:**
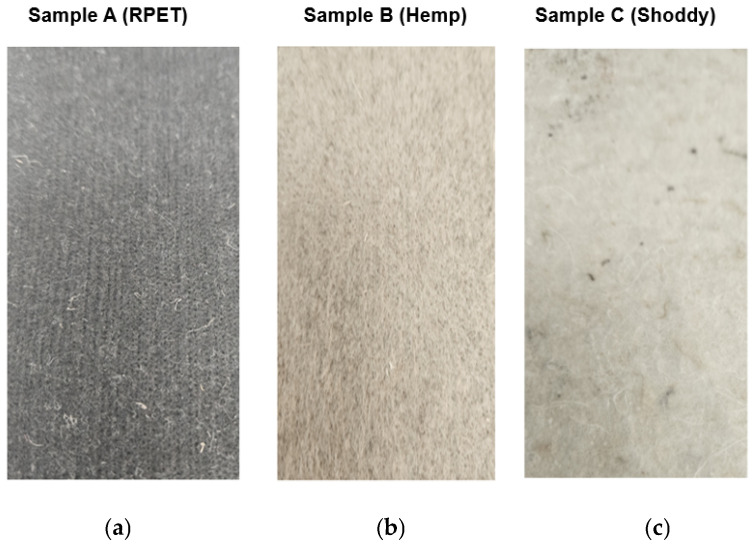
Footbed materials: (**a**) RPET; (**b**) hemp; and (**c**) shoddy.

Three different needle-punched nonwoven footbed mats were produced from RPET, hemp, and shoddy fibers (Figure 1). The length and fineness of the RPET fibers were 40 mm and 2.2 decitex, respectively. The length and fineness of the hemp fibers were 68 mm and 3.2 decitex (cottonized). For the shoddy fibers, there was no definite fiber length or fineness, as it was a mixture of different waste fibers. The needle-punching process parameters for producing the RPET, hemp, and shoddy footbeds were kept constant: feeding speed 0.74 m/min; depth of needle penetration 6 mm, 7 mm; stroke frequency 196, 198 stroke/min, and output speed 1.30 m/min.

### 2.2. Environmental Accelerated Weather Testing

The QUV accelerated weathering testing chamber (from Q-Lab, Westlake, OH, USA) was used to simulate accelerated environmental degradation of the footbed materials. This instrument simulates the effects of natural sunlight and reproduces the damage caused by weathering elements such as sunlight, rain, and dew. The footbed materials were exposed to fluorescent ultraviolet (UV) radiation from UVA-340 lamps (Q-Lab, Westlake, OH, USA), as per the ISO 4892-3:2024 standard [15]. Furthermore, the footbed materials were exposed to water in the UV weathering chamber to reproduce the weathering effects that occur when materials are exposed in actual end-use environments to daylight, or daylight through window glass simulating rain or dew conditions. The accelerated weathering test is carried out in two steps: Step 1—Exposure to UV light at 0.79 W/m^2^ at 60 °C for 8 h; Step 2—Condensation at 50 °C for 4 h. The accelerated weathering test was performed for 360 h. The footbed materials were removed after every 120 h of UV exposure for further characterization. After that, the samples were subjected to 240 h and 360 h of UV exposure and subsequent characterization.

To estimate the exposure time of the footbed materials in the accelerated weathering chamber, the following assumptions were made: The average solar radiation in South Africa is 16 MJ/m^2^/day [16]. Therefore, in 230 days, the total radiation would be 3680 MJ/m^2^. Of this total, 6.3% (231.8 MJ/m^2^) constitutes UV A type radiation (UVA), which is responsible for the UV photo degradation of materials. To estimate the test time, the expected radiation (231.8 MJ/m^2^) was divided by the daily radiation supplied by the equipment (14.4 MJ/m^2^). Therefore, the footbed materials exposed in a QUV accelerated weathering for 120 h are approximately equivalent to 55–60 days of real conditions in South Africa.

### 2.3. Aerobic Biodegradability in Soil and Compost Conditions

**Figure 2:**
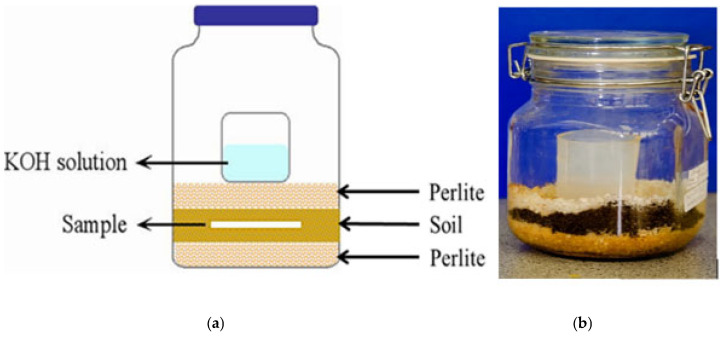
(**a**) A schematic diagram of the biodegradation set-up; (**b**) biometer flask respirometric system used for studying the biodegradation of footbed materials in soil conditions.

Aerobic biodegradability in soil and compost was tested for the footbed material by measuring CO_2_ evolved during the degradation process by using ASTM D5338-21, ASTM D5988-18, and ISO 17556-19 standards [17,18,19]. Figure 2 shows the biometer flask respirometric system used for the soil biodegradation tests under agricultural soil conditions at 25 ± 2.0 °C. For the soil biodegradation test, agricultural soil was obtained from Garden Master, Pretoria, South Africa. The soil was passed through a sieve with a mesh of <0.8 cm to achieve a uniform particle size for the biodegradation study. The carbon dioxide (CO_2_) production from soil and compost biodegradation was conducted in a 1 L biometer-respirometric apparatus (Figure 2). A total of 100 g of the sieved agricultural soil was placed on top of 20 g of perlite, wetted with 20 mL of water, followed by an upper layer of 20 g of perlite, and again wetted with 20 mL of water. This perlite arrangement helps to maintain uniform aeration and humidity in the soil or compost medium, and the elimination of noise in the test results. A total of 40 mL of 1.0 M potassium hydroxide (KOH) solution was placed on the upper layer of the compost mixture in an airtight respirometer apparatus to trap the CO_2_ evolved from the compost inoculum, as shown in Figure 2. The CO_2_ emission was tested in three replicates. A detailed description of the soil biodegradation test is reported elsewhere [13]. This test was performed for RPET as an example to compare with the environmental degradation results. Table 1 shows the physical–chemical properties of the compost and soil obtained.

**Figure 3:**
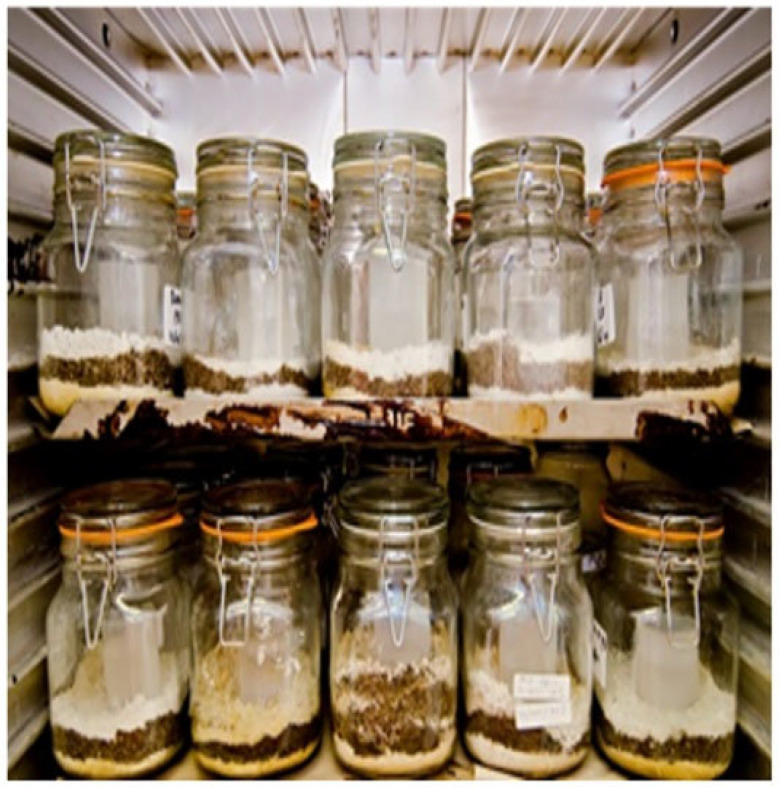
Soil and compost biodegradation testing at laboratory conditions.

For the compost biodegradation test, 3-month-old well-aerated compost derived from a mushroom farm consisting of a mixture of straw, hay, mulch, and chicken manure was used (Figure 3 and Table 1). The compost inocula were screened to less than 10 mm by sieving, and any large inert items were manually removed (pieces of glass, stone, wood, etc.) for biodegradation testing of the footbed materials as per ASTM D6400 standard [20]. As shown in Figure 3, the compost biodegradation test biometer flasks were incubated in the dark at a constant temperature of 58 ± 2 °C. All the test samples were tested in three replicates, similar to the soil biodegradation test. A detailed description of the compost biodegradation test is reported elsewhere [14].

### 2.4. Determination of CO_2_ and Evaluation of Percentage Biodegradation

The biodegradation of the footbed material (RPET) was measured by trapping the emitted CO_2_ in the potassium hydroxide (0.1 N KOH) solution, as shown in Equation (1). The degradation of the footbed material due to the action of microorganisms results in CO_2_ emission. As the gaseous emitted CO_2_ reacts with KOH [13,14],

(1) $$2KOH+{CO}_{2}\to K_{2}CO_{3}+H_{2}O$$

With the reaction end product (soluble K_2_CO_3_), Barium Chloride (1N BaCl_2_) was added, and an insoluble Barium Carbonate (BaCO_3_) was formed as a cloudy white precipitate (Equation (2)). Phenolphthalein was used as an indicator. After titrations, each beaker was washed and refilled with standard 0.1 N KOH solution (Equation (3)). A known biodegradable material, microcrystalline cellulose, was used as a positive control, and all the tests were carried out in triplicate.

(2) $$K_{2}CO_{3}+{BaCl}_{2}\to BaCO_{3}+2KCl\;$$

(3) $$KOH+HCl\to KCl+H_{2}O\;$$

### 2.5. Characterization

#### 2.5.1. Fourier-Transform Infrared Spectroscopy (FTIR)

The FTIR analysis was performed on the footbed materials before and after environmental biodegradation by using a PerkinElmer IR spectrometer (Shelton, CT, USA) with 32 scans and processed with software. After 120 h of QUV environmental weathering, the footbed materials were analyzed for spectra by comparison with a reference library. Similar procedures were repeated for 240 and 360 h of QUV exposure.

#### 2.5.2. Differential Scanning Calorimetry (DSC)

The DSC analysis was carried out by using DSC-8500 (PerkinElmer, Branford, CT, USA) to determine melting and cold crystallization temperatures, and enthalpies (ΔH’s). It was performed by using nitrogen gas, and the temperature range was −40 °C to 200 °C. Each footbed material underwent three successive scans of heating, cooling, and heating at a rate of 10 °C/min. Melting and heat of enthalpy of the footbed material measured before and after 120, 240, and 360 h of QUV exposure were reported.

#### 2.5.3. Thermal Gravimetric Analysis (TGA)

The thermal properties of the test samples were evaluated using TGA (Q600 TA Instruments, New Castle, DE, USA). The samples (10–20 mg) were analyzed over a temperature range of 30 to 800 °C at a heating rate of 10 °C/min by using a nitrogen gas atmosphere. The thermal properties of the test samples were measured before and after 120, 240, and 360 h of the environmental degradation test to determine the changes in onset, derivative degradation, maximum degradation temperature, and residue % (by weight) after 600 °C.

#### 2.5.4. Thickness and Area Weight

The thickness of the footbed materials was measured according to the European Disposables and Nonwoven Association (EDANA) standard, WSP 120.6 (05) [21]. The area weights of the footbed materials (g/m^2^–or gsm) were measured according to ASTM D3776M-20 by using an electronic balance [22].

#### 2.5.5. Tensile Strength and Abrasion Resistance

Tensile strengths were measured on a Titan tensile tester according to ISO 13934-1 standard [23]. Strip tests were conducted, which used the following testing parameters: fabric width 50 mm; gauge length: 120 mm; traverse or stretching speed 100 mm/min. It was measured in both machine direction (MD) and cross direction (CD).

Abrasion resistance of the footbed materials was tested on the Martindale abrasion tester (Rock Hill, SC, USA) as per the ISO 12947-3:1998 standard [24]. The mass loss, pilling intensity, material breakage, and number of abrasion resistance cycles were reported and compared with the standard photographic standards.

## 3. Results and Discussion

### 3.1. Visual Appearance After UV Exposure

**Figure 4:**
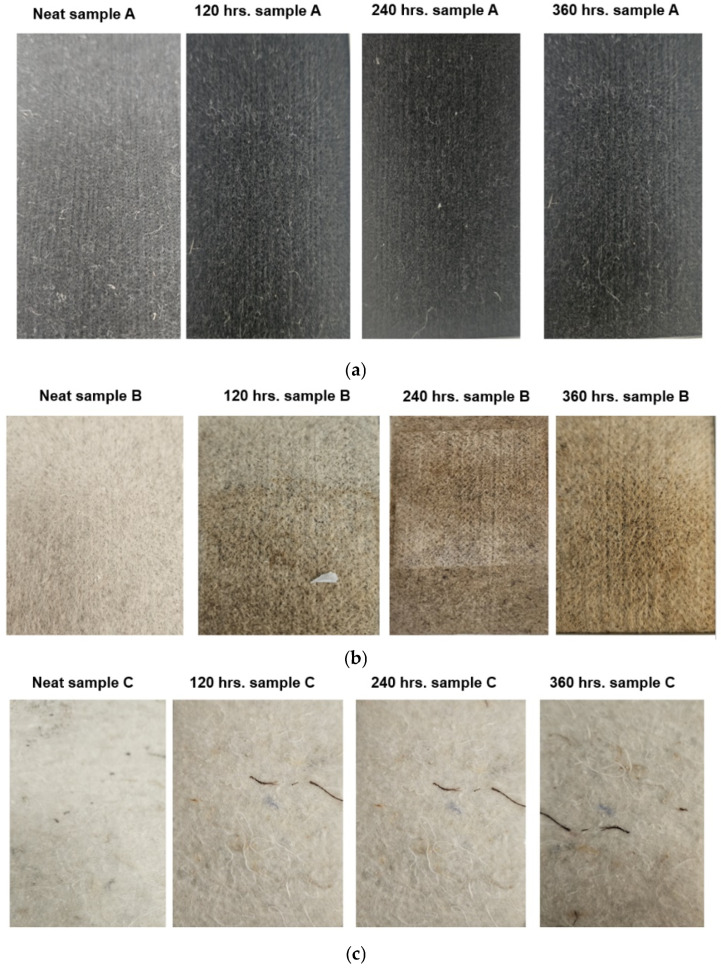
(**a**). RPET original (neat) and RPET exposed to 120, 240, and 360 h in the QUV weathering chamber. (**b**). Hemp original (neat) and hemp exposed to 120, 240, and 360 h in the QUV weathering chamber. (**c**). Shoddy original (neat) and shoddy exposed to 120, 240, and 360 h in the QUV weathering chamber.

The visual appearance of the footbed materials after 120, 240, and 360 h of exposure in the QUV weathering chamber is depicted in Figure 4a–c. No significant changes in the RPET footbed material color were observed after QUV exposure, except that it was a bit darker in color (Figure 4a). RPET is a man-made or synthetic fiber, and it is able to withstand QUV exposure. Significant color changes were observed in the hemp footbed materials after QUV exposure, with color changing from an off-white in the case of neat hemp to a light brown (120 h exposure), dark brown (240 h exposure), and yellow color (360 h exposure), as shown in Figure 4b. Since hemp is a natural fiber, after exposure to UV was degrading the fibers, a color change was observed. Furthermore, the structure of the fiber and the presence of chemical groups that are actively exposed to photon flux and oxidative degradation may further contribute to the color change. For the shoddy footbed materials, no significant changes in color were observed after different QUV exposure, as it was a mix of different synthetic fibers and able to withstand UV exposure, as shown in Figure 4c.

### 3.2. FTIR

**Figure 5:**
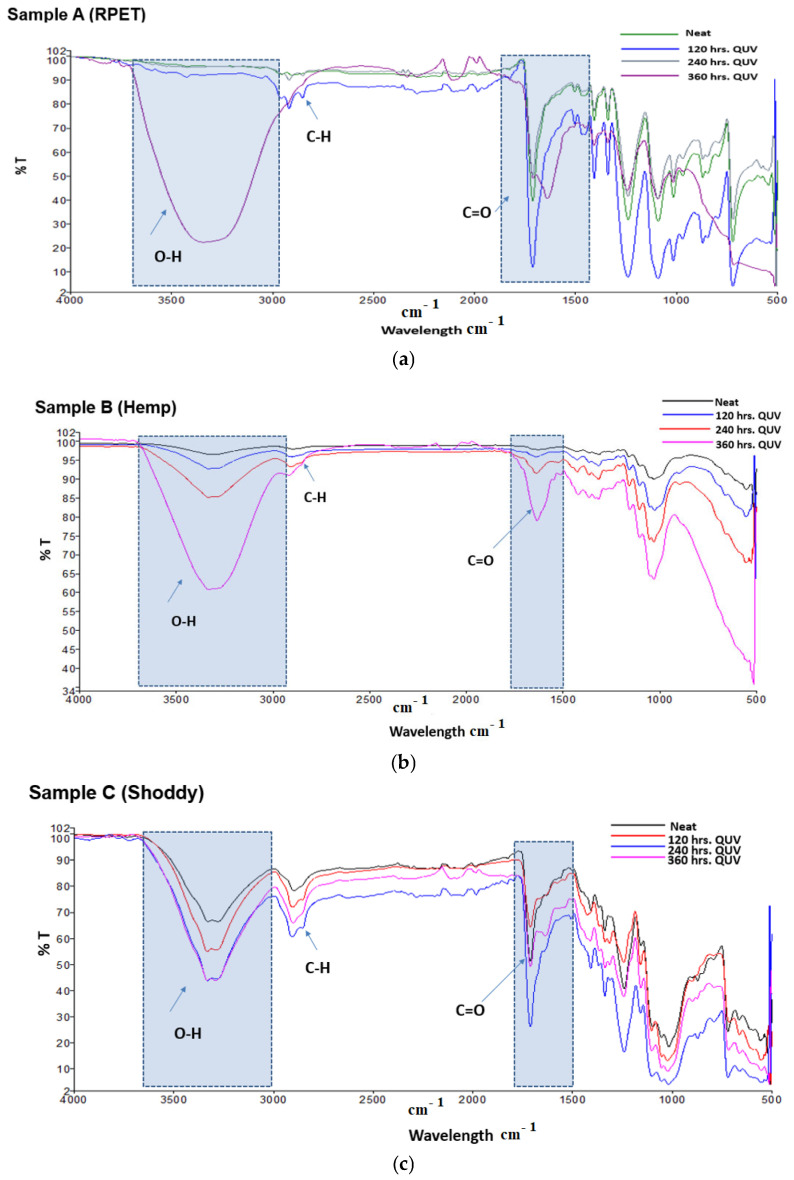
(**a**). FTIR scan of the neat (original) RPET footbed materials and those subjected to different exposure times in the weathering test chamber. (**b**). FTIR scan of the neat (original) hemp footbed materials and those subjected to different exposure times in the weathering test chamber. (**c**). FTIR scan of the neat (original) shoddy footbed materials and those subjected to different exposure times in the weathering test chamber.

The FTIR scans of the RPET footbed materials after exposing them to 120, 240, and 360 h in an environmental weathering chamber are shown in Figure 5a. There were no significant changes in the RPET’s degradation pattern as compared to the neat footbed material after 120 and 240 h of QUV exposure. RPET undergoes hydrolytic abiotic degradation after 360 h and makes the sample more fragile. RPET was severely affected in the functional groups after 360 h, where there was a significant increase in the absorbance of 3000–3600 cm^−1^ hydroxyl group (OH) and absorbance of 1650 cm^−1^ carbonyl keto and ester groups. These increases in the OH and carbonyl oxygen (C=O) functional groups at 1600–1800 cm^−1^ indicate increased hydrophilicity, which facilitates abiotic and microbial attack. The maximum UV exposure hours (360 h) equate to ±180 days. The UV degradation of the RPET with the maximum exposure hours can be attributed to the high ratio of aliphatic compounds as compared to aromatic compounds [25,26].

The FTIR results indicate that hemp undergoes abiotic degradation after 120 h, with a more pronounced effect observed after 240 and 360 h of QUV exposure (Figure 5b). As a result, the footbed materials are fragile and cause the fragmentation process due to the synergistic effect of chemical and hydrolytic degradation (Figure 5b). Furthermore, fragmentation may be due to the photolytic degradation and photo-oxidation process, which mainly occur in the amorphous region of hemp fibers as they are susceptible to oxygen [27,28].

The FTIR results in Figure 5c indicate that the shoddy footbed materials undergo abiotic degradation after 240 h of QUV exposure, influenced by relative humidity, temperature, and UV light. The footbed materials undergo hydrolytic chain breakdown into water-soluble compounds like OH, aldehyde group (CHO), carboxyl group (COOH), and C=O (Figure 5c). Furthermore, another factor may be due to the chain scission reactions and formation of more radicals that can either combine to initiate photo-oxidation, resulting in footbed material degradation [29,30].

### 3.3. DSC

**Figure 6:**
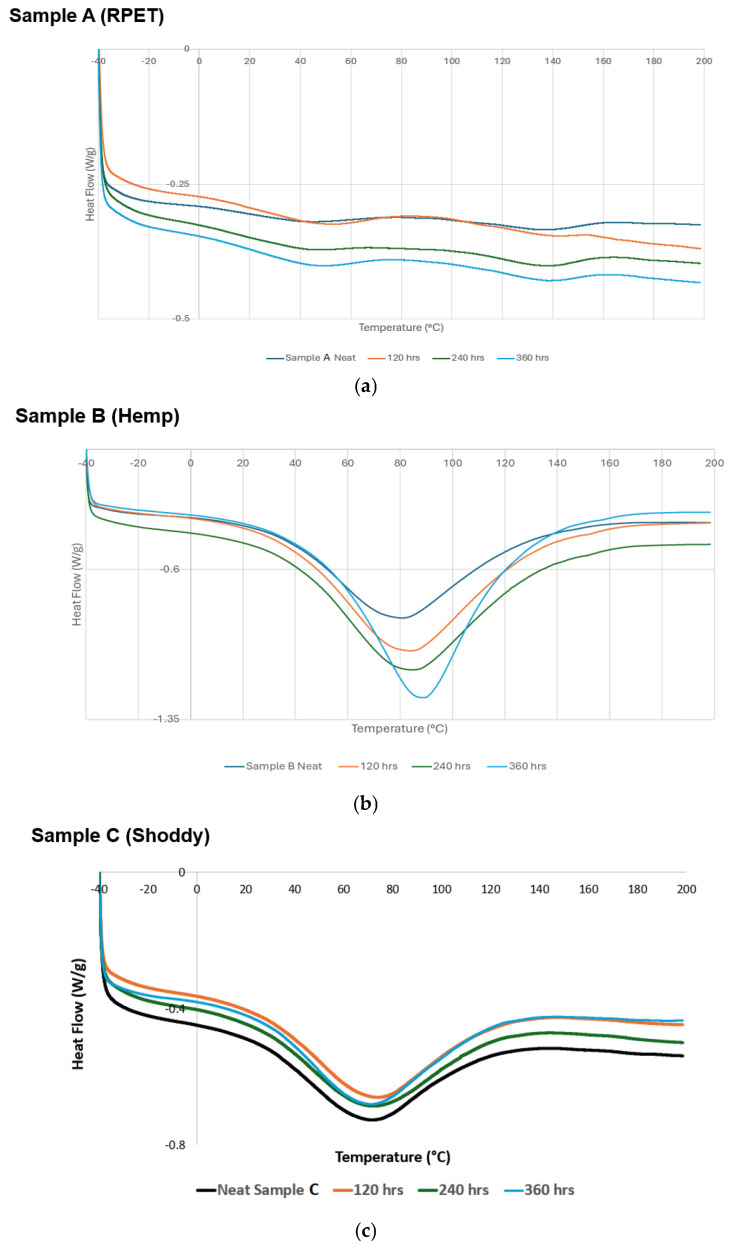
(**a**) DSC scans of the neat (original) RPET footbed materials and those subjected to different exposure times in the weathering test chamber. (**b**) DSC scans of the neat (original) hemp footbed materials and those subjected to different exposure times in the weathering test chamber. (**c**) DSC scans of the neat (original) shoddy footbed materials and those subjected to different exposure times in the weathering test chamber.

The DSC results of the different footbed materials, which were exposed to 120, 240, and 360 h in the QUV weathering chamber, are shown in Table 2. Two melting peaks were observed for RPET, indicating the presence of amorphous and crystalline regions. The first exothermic peak is the cold crystallization due to the ordering of the amorphous region forming into crystals as a result of heating. The second melting temperature is due to the endothermic reaction resulting in the breakdown of the crystalline regions [31]. This trend was observed for all the RPET footbed materials exposed to different hours in the weathering chamber. There is a slight increase in the Tm (cold crystallization) of RPET as compared to the 0 and 120 exposure hours, which can be attributed to the formation of more amorphous regions because of UV exposure, causing chemical degradation in the RPET materials [32]. The heat of enthalpy (Hm) for RPET does not follow any trend. The DSC scans of the neat (original) RPET footbed materials and exposing them to different exposure hours in the weathering test chamber are shown in Figure 6a. There were minor thermal transitions shifts on the RPET footbed materials after QUV exposure, which requires less heat of enthalpy and affects the crystalline part. These results indicate that environmental abiotic factors are influencing the degradation of RPET test samples [26].

For the hemp footbed materials exposed to 120, 240, and 360 h in the weathering chamber, there was thermal degradation happening with exposure to heat instead of melting, as observed in the case of RPET. More pronounced degradations were observed with increasing exposure hours, and similar trends were observed for Hm. Figure 6b shows that a major thermal transition shift occurred after 360 h of QUV exposure. These results aligned with the FTIR results, indicating that abiotic synergistic effects of physical and chemical influences on the degradation [28].

The DSC results indicate that the shoddy footbed materials melted around 70 °C with a slight variation, as these materials were a mixture of different fibers (Figure 6c). There are no changes in the environmental degradation pattern. The Hm for the shoddy footbed materials does not follow any trend due to blending of different fibers.

### 3.4. TGA

**Figure 7:**
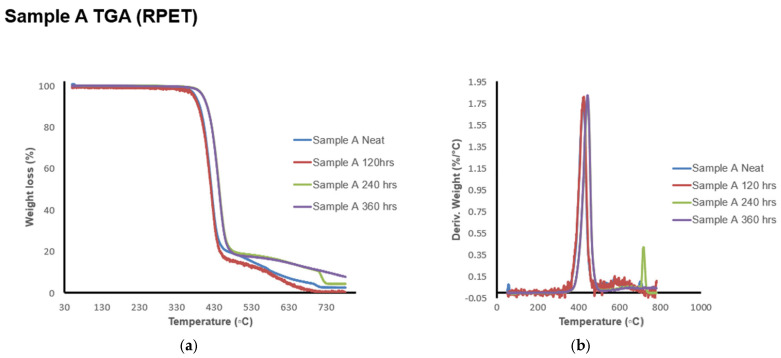
(**a**). TGA thermograms of the neat (original) RPET footbed materials and those subjected to different exposure times in the weathering test chamber; (**b**). the derivative thermogravimetric (DTG) curves of the RPET footbed materials and those subjected to different exposure times in the weathering test chamber.

The TGA results of different footbed materials, which were exposed to 120, 240, and 360 h in the QUV weathering chamber, are shown in Table 2. The TGA results for RPET show minor changes in the Tonset 5% weight loss (T_5%)_ temperature and maximum degradation temperature (T_max_) after QUV exposure (Figure 7a,b). These results indicate that environmental factors influence the primary chain scission of the RPET footbed materials. After QUV exposure, the decrease in RPET Tonset could be due to the polymeric chain scission in the amorphous region by the photo-oxidation process. During this degradation process, the hydrolytic degradation of hetero chain functional groups like carboxylic acids, aldehyde, and ester groups is sensitive to thermal degradation. On the other hand, there are no significant changes in the maximum degradation temperature, which could be due to the RPET bulk cross-linked terephthalate [26,31,32]. In the case of the hemp test sample, Tonset and Tmax showed significant changes after QUV 120, 240, and 360 h of exposure. This reduction in Tonset and Tmax had occurred due to degradation of lignin, hemicellulose, and cellulose components of hemp [33]. The main decomposition temperature ranges of hemicellulose and lignin occurred between 100 and 120 °C and 120 and 150 °C, respectively, whereas that of cellulose would have occurred at 200–350 °C [33]. In the case of the shoddy footbed test sample, a slight degradation was observed on the Tonset and Tmax after 120, 240, and 360 h of QUV exposure. This degradation behavior is similar to the RPET test sample, where minor thermal degradation could be due to photo-oxidation in the amorphous region. This may be attributed to gradual degradation. The DSC and TGA results indicate that hemp footbed materials have a higher rate of abiotic degradation as compared to RPET and shoddy footbed materials (Table 2, Figure 7a,b).

### 3.5. Biodegradation

The aim of biodegradation is to evaluate the ultimate biodegradability in terms of CO_2_ production of the material in soil and compost conditions. With the growing concern regarding the use of different polymeric materials, sustainable issues, and their environmental footprint, we have tested one of the most widely used polymeric materials, RPET, for biodegradation tests in soil and compost conditions for 110 days. This study was mainly focused on measuring the ultimate CO_2_ biodegradation rather than the primary degradation testing of weight loss and disintegration testing. During the biodegradation testing, no visual particles were observed after 30 days, where the results indicate that during the mineralization process, the polymeric chain broke into smaller molecular compounds (monomer, dimers, and oligomers) by enzymatic steps, and was followed by mineralization into final end-products like CO_2_, H_2_O, and new microbial biomass. During these conditions, the test samples became more fragile after 30 days due to the growth of natural microorganisms present in compost and soil conditions, and were not visually distinguishable to take samples for microscopy analyses. The RPET test sample showed a higher biodegradation rate under composting conditions as compared to the soil medium. These results could be due to the presence of thermophilic bacteria and fungi rather than mesophilic bacteria present in soil conditions.

**Figure 8:**
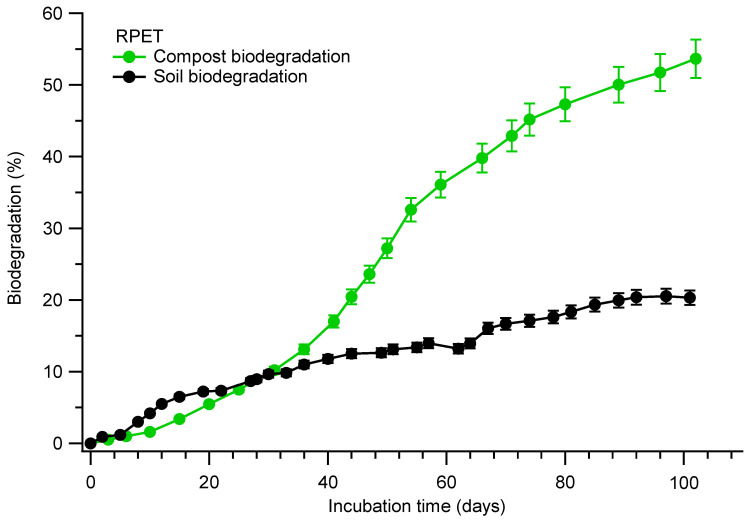
CO_2_ biodegradation of RPET in soil and compost conditions.

The CO_2_ biodegradation results of one of the footbed materials, i.e., RPET, carried out under soil and compost conditions are depicted in Figure 8. RPET soil biodegradation results indicate that after a logarithmic phase, reaching 13% biodegradation within 35 days. After that, there was a slow rate of biodegradation, reaching 20% biodegradation in 110 days. On the other hand, the compost biodegradation results showed a higher rate of biodegradation with an exponential phase reaching 59% within 110 days. These results indicate that the microbial communities present in compost medium greatly enhance the biodegradation of RPET than soil conditions [13].

Biodegradation of RPET in soil and compost involves microbial assimilation of low-molecular-weight compounds during the chain scission reaction of the RPET test samples, where ester bonds (-COOR) were broken down through chemical and microbial hydrolysis processes. During the abiotic QUV accelerated weathering testing, the formation of functional groups, such as (-OH) and (-COOH), and changes in thermal properties, such as Tonset, Tmax, melting temperature, and heat of energy, were observed. During the biodegradation process, the release of intermediate molecules of low-molecular-weight compounds (-COOR, -OH, and (-COOH)) was metabolized by natural microorganisms present in soil and compost conditions. The abiotic and biotic degradation processes of test samples and their degradation mechanism were confirmed by the FTIR, TGA, DSC, and CO_2_ results [28,29,30,34,35,36]. Future research may focus on studying long-term degradation testing (>1 year) in soil and compost conditions.

### 3.6. Physical and Performance Properties

The properties of the different footbed materials are shown in Table 3. There is a slight difference in the area weight and thickness of the selected footed materials, as these fibers were sourced from different suppliers. The tensile strength range of the materials was 50.74–851.44 N. The weight loss range after abrasion resistance was 0.016–0.014%. The tensile strengths in both machine and cross direction were highest for RPET, followed by hemp and then shoddy materials. Since hemp is a natural fiber, there are variations in fiber properties, which result in less strength as compared to RPET. The shoddy footbed material is a mixture of different fibers, resulting in the lowest performance properties among the selected materials. RPET withstands 35,000 abrasion cycles, showing severe pilling, no breakage in the materials, and the highest mass loss amongst the selected materials [1]. It was followed by hemp, withstanding fewer abrasion cycles (5000), showing severe pilling, breakage in the material, and less mass loss as compared to the RPET. The least performance properties were shown by shoddy materials, only lasting 1000 abrasion cycles, with sample breakages during testing, and no further testing was possible. It shows that RPET is a better material in terms of its performance properties compared to hemp and shoddy materials, as the original source of it is from synthetic polymers. Among the materials studied, RPET showed superior mechanical stability and moderate biodegradation, suggesting its suitability for semi-durable applications requiring both performance and partial environmental degradability.

## 4. Conclusions

The environmental degradation and biodegradation of RPET, hemp, and shoddy footbed materials were studied. RPET undergoes hydrolytic abiotic degradation after 360 h and makes the material more fragile. Hemp footbed materials undergo abiotic degradation after 120 h, resulting in the fragmentation process as a synergistic effect of chemical and hydrolytic degradations. The DSC results indicate that RPET undergoes a slight thermal transition under abiotic degradation after 360 h, indicating that environmental abiotic factors influence the degradation of RPET. The TGA results indicate that environmental factors influence the primary chain scission on RPET footbed materials. The biodegradation test results showed that the RPET footbed materials achieved 20% and 59% degradations in soil and compost conditions, respectively, in a 3-month time frame. The tensile properties of RPET were the highest, followed by hemp and shoddy materials. There was a significant difference in abrasion resistance of the RPET, hemp, and shoddy materials, with the highest abrasion resistance for RPET, followed by hemp, and then the least was for shoddy material.

RPET demonstrated superior mechanical stability and biodegradation behaviors, indicating that compost medium is a suitable condition for enhanced biodegradation as compared to soil conditions. Furthermore, these findings imply that the design of materials is appropriate for semi-durable applications that meet both performance with added advantages of partial environmental degradability.

## Figures and Tables

**Table 1:** Physical–chemical properties of the compost and soil used in this study.

Analysis	Compost	Soil
Total dry solids (%)	55	80.9
Volatile solids (%)	53	25.5
pH of solution	7.1	7.2
Total organic carbon amount (%)	10.6	3.4
Total nitrogen amount (%)	0.9	0.12
C/N ratio	11.7	28.3

**Table 2:** DSC and TGA results of the different footbed materials exposed to 120, 240, and 360 h in the QUV weathering chamber.

		DSC		TGA	
Footbed Materials	QUV Duration (Hours)	T_m_ (°C)	H_m_ (J/gm)	T_5%_ (°C)	T_Max_ (°C)
Sample A RPET	0	42.78, 136.67	3.48, 3.95	380.22	425.57
	120	42.22, 138.62	2.09, 1.16	375.83	425.85
	240	46.34, 137.65	3.98, 2.91	380.86	424.42
	360	46.85, 139.55	4.01, 3.04	379.60	423.02
Sample B Hemp	0	80.79 *	180.62	145.50	342.69
	120	84.01	214.30	120.50	329.60
	240	85.76	226.34	115.50	315.50
	360	88.78	255.44	104.60	296.70
Sample C Shoddy	0	70.58	69.81	360.01	421.62
	120	73.10	88.61	359.30	419.56
	240	70.92	78.06	358.87	420.30
	360	72.15	90.85	357.17	419.60

* Thermal degradation.

**Table 3:** Physical and performance properties of different footbed materials.

Properties	Sample A: RPET (100%)	Sample B: Hemp (100%)	Sample C: Shoddy
Area weight, (g/m^2^)	445	470	410
Fabric thickness, (mm)	2.69	2.02	2.25
Tensile strength—Machine direction, (N)	851.44	395.01	85.10
Tensile strength—Cross direction, (N)	499.97	152.94	50.74
Abrasion resistance cycles	@35,000 revolutions Severe pilling No specimen breakage Shade change—Rating 4	@5000 revolutions Severe pilling Specimen breakage was detected, and the test stopped. Shade change—Rating 4	@1000 revolutions Severe pilling Specimen breakage was detected, and the test stopped. Shade change—Rating 4
Mass loss average (mg)	71.74	65.82	-

## Data Availability

The raw data supporting the conclusions of this article will be made available by the authors upon request.

## Abbreviations

The following abbreviations are used in this manuscript:

FTIR	Fourier-Transform Infrared Spectroscopy
DSC	Differential Scanning Calorimetry
TGA	Thermogravimetric Analysis
RPET	Recycled polyester
UV	Vltraviolet
